# How can clinical immunology laboratories contribute to the management of severe COVID-19 cases in limited resource contexts?

**DOI:** 10.4102/ajlm.v9i1.1282

**Published:** 2020-11-02

**Authors:** Brahim Admou, Abdelhamid Hachimi, Mohamed Abdenasser Samkaoui

**Affiliations:** 1 Center of Clinical Research, Faculty of Medicine and Pharmacy, Mohamed VI University Hospital, Cadi Ayyad University, Marrakech, Morocco; 2 Department of Intensive Care, Faculty of Medicine and Pharmacy, Mohamed VI University Hospital, Cadi Ayyad University, Marrakech, Morocco; 3 Department of Anesthesiology and Intensive Care, Faculty of Medicine and Pharmacy, Mohamed VI University Hospital, Cadi Ayyad University, Marrakech, Morocco

## Introduction

Since its appearance, the coronavirus disease 2019 (COVID-19) that is caused by severe acute respiratory disease coronavirus 2 (SARS-CoV-2), has been responsible for severe respiratory disease similar to diseases commonly associated with the coronaviruses such as severe acute respiratory syndrome and Middle East respiratory syndrome; COVID-19 frequently requires hospitalisation in the intensive care unit and has had a high mortality rate.^1^ The severity of COVID-19 is generally stratified as asymptomatic, mild (without signs of severe or critical illness), severe (with signs of breathing difficulties) or critical (with signs of respiratory failure, shock and multiple organ failure).^2^

The contribution of the medical laboratory to the categorisation of COVID-19 clinical forms is not yet well defined.^3^ After confirmation of infection by real-time polymerase chain reaction, biochemical and haematological analyses provide fundamental biological parameters for determining disease severity. Similarly, the clinical immunology laboratory could play an important role in elucidating diverse immunological abnormalities associated with the disease. In particular, immunological testing could better categorise the severe and critical forms of COVID-19, and subsequently assist treating physicians during the entire course of therapy. The purpose of this opinion article is to highlight the position of the immunology laboratory in the management of severe and critical COVID-19 cases, specifically in countries with limited resources.

## COVID-19 and cytokine storm

COVID-19 is marked by an overproduction of pro-inflammatory cytokines and chemokines, mainly interleukin (IL)-6, IL-10, tumor necrosis factor (TNF)-α, IL-1β, IL-2, IL-10, interferon-γ and monocyte chemoattractant protein-1.^4^ Other biological abnormalities may predict the severity or progression of the disease, such as abnormal coagulation activation, leukopenia, high levels of C-reactive protein, ferritin, D-dimers, aminotransferase, lactate dehydrogenase and creatin kinase.^3,5^ Elevated levels of pro-inflammatory cytokines have been shown to characterise severe lung infection, which manifests as respiratory distress, multiple organ failure and adverse outcomes in SARS-CoV-2 infection.^6,7^ In addition, IL-6, IL-10, and TNF-α levels significantly increase during infection and drop during recovery,^8^ suggesting that the intensity of the cytokine release correlates with disease activity. Interestingly, IL-6 may be a marker for monitoring serious COVID-19 cases.^9^

Moreover, simultaneous high levels of IL-6 and D-dimers have been shown to be narrowly associated with severe forms of the disease in adult patients. Therefore, measuring them in combination allows for greater specificity and sensitivity for predicting severe cases of COVID-19.^3^

## COVID-19 and T cell impairment

Deregulation of the immune response, particularly T lymphocytes, appears to be strongly linked to the pathology of COVID-19^10^; direct infection of lymphocytes by SARS-CoV-2 has been proposed as a cause for acute lymphocyte decline.^3,11^ Lymphocytes express the receptor angiotensin-converting enzyme 2, which has been suggested to be the primary receptor targeted by SARS-CoV-2.^12^

Moreover, altered T lymphocytes could be an important factor in worsening symptoms in patients, which makes lymphopenia a relevant marker of disease severity, hence the need for intensive care unit admission.^11^

It has been demonstrated that the severe respiratory syndrome that manifests due to COVID-19 is characterised by cluster of differentiation (CD)4 and CD8 T lymphopenia, correlating with disease severity.^4,13^ On the other hand, COVID-19 patients who require intensive care unit hospitalisation have significantly higher levels of IL-6, IL-10 and TNF-α, with lower levels of CD4 and CD8 T cell counts,^8^ with which levels of cytokines and T cells are inversely correlated.^6,8^ Moreover, because T lymphocytes are generally crucial for amortising exaggerated natural immune reactions against viral infection, T cell defects can lead to worsening inflammatory responses during COVID-19, whereas restoring the number of these lymphocytes can improve them.^6^

In accordance with this hypothesis, it has been shown that 4–6 days following the onset of infection, T lymphocyte counts drop to their lowest level, whereas cytokine levels reach their maximum. Conversely, the restoration of the number of T lymphocytes is associated with a decrease in serum levels of various cytokines, such as IL-6, IL-10 and TNF-α.^6^ Owing to the cytokine storm, and other immunological predictors of severity of SARS-CoV2 infection, these may also be helpful in choosing anti-inflammatory drugs, especially corticosteroids for their potential benefit in the reduction of inflammation-induced lung damage in severe COVID-19 patients.^1^

## COVID-19 and immunological investigations

Having shown that the most relevant immunological parameters include inflammatory cytokines and T lymphocyte subpopulations, the clinical immunology laboratory is positioned to be important for assessing COVID-19 patients, especially for categorising, or even predicting severe cases.^2,10^ Cytokine levels, especially that of IL-6, can be individually measured on immunoassay analysers, whereas other cytokine profiles can be explored using either enzyme-linked immunosorbent assays or other specific biotechnologies like Luminex^®^, 200^™^ (or multiplex) (Luminex^®^, Austin, Texas, United States) and flow cytometry systems, which allow for a large-scale quantitative measurement.^14^ The assessment of the main T cell subsets, such as CD3^+^, CD4^+^ and CD8^+^, requires only a simple phenotyping procedure conductible on a mini-cytometer, which is largely available worldwide even in limited-resource context laboratories, thanks to HIV management programs. More developed flow cytometry platforms allow for comprehensive phenotyping assays, enabling the investigation of naive, memory and regulatory T cells; in severe COVID-19 patients, early data on T cell subpopulation abnormalities show increased naive helper T cells and decreased memory helper T cells, associated with a lower number of regulatory T cells.^10^

Using a receiver operating characteristic curve to compare fatal and recovered COVID-19 cases, Xu et al.^2^ considered 559 cells/*µ*L, 235 cells/*µ*L, and 104 cells/*µ*L of CD3^+^, CD4^+^T, and CD8^+^ T cell subsets as warning values, below which there was a significantly higher risk of in-hospital death.^2^ Indeed, there is a need for regional data analyses before determining cut-off values of these T cell subsets. However, T cell lymphopenia might be accentuated by possible primary or acquired immune deficiency conditions, potentially revealed by COVID-19, and must then be considered first when interpreting cell phenotyping values and when managing patients as well.^15^

### Conclusion

For SARS-CoV-2 infection, alongside other clinical and biological parameters, the measurement of inflammatory cytokines, mainly IL-6, as well as CD4+ and CD8+ T cell assessment, should be systematically planned during management of the disease. These markers could be useful for identifying severe cases requiring prompt admission to intensive care units, and for monitoring patient disease progression. These investigations are within the reach of almost all clinical immunology laboratories in the world. Close collaboration between immunologists and physicians is essential for effective global efforts against this highly threatening pandemic.
